# Hospital and Institutional News

**Published:** 1915-11-20

**Authors:** 


### HOSPITAL AND INSTITUTIONAL NEWS.

### ST. BARTHOLOMEW'S HOSPITAL.

We congratulate the governors of St. Bartholomew's Hospital on the fact that the Lord Chamberlain, the Right Hon. Lord Sandhurst, G.C.I.E., G.C.S.I., has resumed his active work as treasurer of St. Bartholomew's Hospital. No one could have worked harder or taken a greater or more useful interest in the welfare and maintenance of the voluntary hospitals than Lord Sandhurst, and we welcome his return to active service with infinite pleasure and the greatest satisfaction. Mr. Acton Davis, who was appointed acting treasurer of this hospital on July 25, 1912, has resigned his appointment. During Mr. Acton Davis's period of office, over three years, a loan of £30,000 to the bank has been repaid, the hospital has now no overdraft at its bankers, and its financial position has so improved as to enable it to have money on deposit with the Union Discount Company of London. Mr. Acton Davis took an active part in obtaining a grant of £10,000 in 1913 from the Trusteees to the Zunz Bequest. Under him material advantage has accrued to the hospital's estates, and matters of considerable magnitude, including the organisation of the management of the Hospital Estates Department, have been successfully carried out. Amongst other matters usefully accomplished under him, we may mention the arrangements under the National Insurance Act, the establishment at the hospital of a tuberculosis dispensary, and an agreement with the London County Council and the Insurance Committee of the City of London for the treatment of in-patients suffering from tuberculosis. Mr. Acton Davis has exhibited great tact and a broad-minded spirit in his guidance of questions relating to the medical schools and to medical education generally. The arrangements with the War Office for the reception of wounded soldiers—1,797 of whom have passed through the wards of the hospital—owe much to the late acting treasurer, who has been particularly happy in his relations with the medical and nursing staffs, and has throughout taken a practical interest in the affairs of the Women's Guild. The experience which Mr. Acton Davis has gained during his term of office cannot fail, we hope, to prove of continuous value to the administration and success of St. Bartholomew's Hospital in the future.

### THE ENLARGEMENT OF THE ROYAL RED CROSS.

Hitherto the decoration known as the Royal Red Cross has been an Order of one class only; and all the recipients of this, the only official nursing decoration, have been on an equality of status within it. By a new Royal Warrant, just issued but dated November 10, several important changes have been introduced which modify the decoration very considerably. It may now be conferred not only upon ladies of the British Royal Family but also upon "the Queens or Princesses of foreign countries who may have specially exerted themselves in providing for the nursing of the sick and wounded of foreign armies and navies." It may also now be awarded on the recommendation of the First Lord of the Admiralty, as well as on that of the Secretary for War as hitherto. It is to be divided into two classes; the first class is to be distinguished by the old initials "R.R.C.," signifying Member of the Royal Red Cross (first class), and A.R.R.C., signifying Associate of the Royal Red Cross (second class). The awards of the first class are not to exceed 2 per cent. and of the second class 5 per cent. of the total establishment. It would be of interest to know how the existing recipients of this decoration are to be classified under the new scheme.

### THE PLANNING OF BASE HOSPITALS IN FRANCE.

Dr. Fred Hall, who left his post as assistant M.O.H. at Derby to take charge of the Manchester Ward in the base hospital in France, which has been built by the Order of St. John of Jerusalem, has given an interesting description of the novel planning adopted. We take the following from the *Manchester Guardian*: "The hospital [according to Dr. Hall's published statement] is planned rather differently from the Government hospitals. The wards are arranged on the outside and the administration buildings in the centre, and all are connected by a covered way—a feature peculiar to this hospital. This covered way deserves special mention, for it contributes in no small way to the ease and comfort with which patients can be transported from one part of the hospital to another. It is a raised wooden platform which connects the separate buildings one with another, and by means of it a badly wounded soldier is taken from the ambulance, placed on a wheeled stretcher, and carried quickly and smoothly right away to his bed. There is no lifting and no jolting such as may occasionally happen when the patient is carried by two orderlies over soft, sandy ground. This covered way also enables us to deal much more expeditiously with a large convoy of wounded and to get them quickly into their beds. On a cold winter's night the least delay in admitting the patients adds greatly to their discomfort." It will be recalled that the whole institution cost £50,000, and that the people of Manchester raised £3,000 to have a ward named after the city.

### A THREE YEARS' DISPUTE FOR NOTHING.

After a dispute extending over a period of three years the Halifax Corporation Health Committee and the local Insurance Committee have arrived at an agreement for the treatment of insured persons suffering from consumption. The Insurance Committee have agreed to pay 7½d. out of the 9d. they receive for every insured person in respect of sanatorium benefits. In return the Corporation is to provide at least fifteen beds at the Shelf Sanatorium, as well as to supply such dispensary treatment as is required for cases needing it. The agreement is for thirty years, but the amount of payment by the Committee to the Corporation is open to revision on March 31, 1917, and at the end of every subsequent five years. It should be added that the above settlement includes an agreement by the Corporation to make a proportionate allowance to the Insurance Committee in respect of every patient for whom the tuberculosis officer and the panel doctor recommend special treatment at another sanatorium. The question of sending patients to sanatoria other than that of the Halifax Corporation was the cause of much dispute, for while the Insurance Committee urged that patients could be sent away on the recommendation of either the tuberculosis officer or the panel doctor, the Health Committee was of opinion that such a course should only be pursued on the recommendation of both doctors. Moreover, the Insurance Committee claimed a share in the management of the sanatorium on the ground that such was the intention of the Act. The Corporation refused to admit this, and both the Insurance Commissioners and the Local Government Board declined to arbitrate. In the meantime the charges of £2 2s. per week for patients at Shelf Sanatorium and of 10s. a week for dispensary treatment proved more than the Insurance Committee was able to pay, so a conference of two sub-committees appointed by each party was held and effected the above settlement, which, by the way, proves that the Corporation's proposals three years ago were more favourable than those offered last year. These unnecessary disputes are deservedly costly.

### THE BRITISH RED CROSS SOCIETY IN SPAIN.

At a meeting held at the offices of the British Chamber of Commerce in Barcelona, with the British Consul-General (Mr. C. S. Smith) in the chair, it was decided to form an Anglo-Spanish Branch of the British Red Cross Society. The Consul-General accepted the post of local president, and a committee was elected, composed of leading members of the British colony in Spain. It is hoped that all the British consular officers in Spain will assist by forming branches in their respective districts; and the scheme received cordial recognition from the Spanish authorities. Mr. Kendall Park is honorary secretary; Sir Arthur Hardinge, the Ambassador at Madrid, is honorary president, and Lady Hardinge is honorary lady president. We wish the new undertaking every success.

### REDUCTION OF SANATORIUM BENEFITS.

The not unexpected announcement has just been made concerning the building of the three new sanatoria by the Metropolitan Asylums Board, to which reference was made in these notes early in the year. It has been decided that no further progress shall be made in the construction of these institutions. As a matter of fact, in one case only have the preliminary stages of building been entered upon. The three sites selected were in the neighbourhood of Godalming, East Grinstead, and Basingstoke respectively, and the sanatoria were to provide accommodation for 232 women and 343 men. The cause of this abandonment is not entirely due to the war, but is owing to the financial position in which the London Insurance Committee finds itself. As is well known, this Committee has been sending insured tuberculous patients to the Downs Sanatorium and to the Northern Hospital, both being institutions under the control of the Metropolitan Asylums Board. It is now announced that the London Insurance Committee will not guarantee after the end of this year to maintain a larger number than 250 patients in these sanatoria. This will mean a very appreciable reduction in the number of patients treated on behalf of the Committee as compared with the figures for 1914. But a noticeable reduction in the admissions has already been taking place during the last two months.

### A TRAGEDY OF INEXPERIENCE.

During the current year a series of lectures on various subjects by different people have been organised by the Chadwick Trustees. Some of them have possessed practical value, and in the whole they have covered much ground. Experience, however, leads us to think that their usefulness and practical value would have been infinitely greater had care been taken to formulate rules for the guidance of each lecturer and to appoint no one to this office unless the trustees knew that he was accustomed to this kind of work, that he had a presence and voice and sufficient practice to render it certain that he would be distinctly heard and clearly understood. On the general question we may point out that in the case of lectures which are illustrated by pictures thrown on a screen it is fatal to entrust such a demonstration to anyone who has not the voice for the part or who may have acquired a habit of keeping his back to the audience during the whole or the greater part of the time when the pictures are on the screen. When a lecturer has not a written MS. and is not a practised speaker the result may prove disastrous. The larger the audience and the keener its interest the more certain is it that much discomfort, culminating in grave dissatisfaction to many present, must result. Such a lecturer often perpetrates a tragedy the effects of which will linger in the memories of his audience in exact proportion to the interest each person present may take in the selected subject.

### THE PROTECTION OF ADVANCED CONSUMPTIVES IN POOR-LAW INSTITUTIONS.

Adverse comments are frequently made by committee-men on the treatment, or, rather, lack of treatment, of advanced cases of consumption. A typical instance is reported from Kettering, and was discussed at a meeting of the Northamptonshire Insurance Committee. The particular circumstances of the case call for no detailed mention, as they are of an order commonly met with. The usual complaint was made that the patient was "left to die," and that no institutional treatment was offered except the Union infirmary. It would be manifestly unfair that room should be found for chronic advanced cases at the expense of the improvable ones, and "sanatoria" are not suitable places for this class of patient. Until special hospital accommodation is provided, it is inevitable that advanced and dangerous cases should find their way into Union infirmaries; indeed, it is the urgent duty of tuberculosis officers and sanatorium sub-committees to exert pressure in this direction rather than to allow such cases to remain at home and to become centres of infection to others. Mr. Handel Booth the other day expressed surprise and indignation that Poor-Law institutions should have to receive insured consumptives; his information on this point is not apparently up to date, or else he would have known that in London, at any rate, such a transference of chronic, advanced cases from sanatoria to infirmaries has been going on for a year or two. The facts here stated prove the urgent obligation placed upon the Local Government Board to revise the regulations and accommodation of all Poor-Law establishments receiving chronic cases of tuberculosis, so as to remove all taint of pauperism from these unfortunate patients. This must be done, and the sooner the better, in the interests of the sufferers and of the public health.

### THE APPEAL OF ABERDEEN ROYAL INFIRMARY.

We notice with sympathy that the front page of last Saturday's issue of the *Aberdeen Free Press* is almost wholly devoted to an appeal on behalf of the Royal Infirmary of that city. Three columns of advertisement begin by a statement in which excerpts from the accounts show that there was a deficit of some £5,000 last year, and conclude with a list of new annual subscriptions and donations. The latter pleasantly prove that half of the above deficit has been subscribed. But it is hoped by December 31 not only to show a clean sheet, but a healthy advance in the regular income afforded by annual subscriptions. There is a brief article in the body of the paper supporting the institution's claims, being in fact the appeal itself rewritten, but no attempt seems to have been made to interest the public in anything but the institution's accounts. We cannot therefore regard the above as a happy example of appeal-drafting; but since the result of this humdrum method has been to bring in £500 of annual subscriptions, besides £2,500 donations, might not the managers of the Aberdeen Royal Infirmary see in this a promise of what *might* be raised if the *work*, departments, and life of their institution were presented, in a less bald fashion, by newspaper articles, lectures, and illustrated pamphlets?

### THE FUTURE OF FARNFIELD REFORMATORY.

The London County Council is proposing to hand over Farnfield Reformatory to the Board of Control with a view to its adaptation and use as a State institution for defectives of dangerous or violent propensities. The number of inebriates now at the reformatory is fifty only, and these will be found accommodation elsewhere. The property is to be taken over from the beginning of next month, for the duration of the war and for a further period of at least twelve months, at a rent which is to be fixed on the basis that the County Council makes neither a profit nor a loss on the transaction. It is hoped to retain some of the staff, but it is proposed to terminate the engagements of the medical officer, Dr. C. F. Williamson, and the two visiting chaplains.

### THE RESURRECTION OF THE SUNDAY FUNDS.

For some years before the outbreak of war it was the fashion to declare that the voluntary hospital system needed increasing support from the State and the municipality, and the relatively stagnant condition of many of the Hospital Sunday Funds were regarded as a proof of declining support. It is hardly necessary to remind our readers that we never endorsed this view, and faced the position of the Sunday Fund by asking how much initiative was really now being expended on this movement, and by asking the clergy to prove that they had not fallen away from their first care. It is, therefore, perhaps worth while to point out that the war has justified our criticism and shown that the voluntary principle is supported as affectionately as ever in this country. Of the voluntary work performed in connection with the war we would not now speak. It is patent to everybody. We would confine ourselves to noting the wonderful way in which the Hospital Sunday Fund movement itself has revived during fifteen months of war. The receipts, far from declining, have, we believe, universally been maintained or increased, and no single fact can be more encouraging to those who are concerned with the future of the voluntary system. A movement in many places stagnant, in many more retrogressive, has sprung again to life. The returns of the Sunday Funds throughout the great cities are a striking proof of this. They show that, whatever else may be vitally affected by the war, the voluntary system is positively drawing sustenance and vitality from it.

### A BRITISH HOSPITAL AT LIMOGES.

The daily Press has recently published a doubtless well-deserved eulogy of a military hospital for French wounded which is established at Limoges and is supported by the Wounded Allies Relief Committee, a British organisation. This hospital, it appears, has 170 beds, and is greatly admired by the French people in the neighbourhood, as well as by the French medico-military authorities, according to a report by Dr. Brend, who has recently inspected it. His report, however, indicates a good many deficiencies of equipment, some of them of a very serious nature; and we think the time is rapidly approaching when the whole question of these voluntary auxiliary hospitals in France and other Allied countries will have to be reconsidered. In favour of them is the important fact that they are tangible evidence of the solidarity and cordial relations of the Allied nations; but when that has been said there is but little else to add.

### SOME DRAWBACKS.

Against the voluntary auxiliary hospitals are several factors. They are always small, therefore wasteful and inefficient, because they want a larger staff in proportion to their capacity than do hospitals of 500 or 1,000 beds; and, being small, they cannot be, and are not in fact, provided with adequate special departments in the way of *x*-rays, bacteriology, and so forth. Moreover, working as they do in connection with a foreign army (and in the case of Limoges at a vast distance from the nearest fighting front), the work that a given equipment and staff can do is less in amount and in quality than could be achieved for our own troops, simply because of the hundred and one variations between the regulations of the two Services. The report in question, though intended to be eulogistic, suggests very forcibly to the initiated that the time has come to drop these isolated and inco-ordinated enterprises, however valuable they may have been hitherto. We do not quite understand Dr. Brend's position either: we were under the impression that he held a whole-time appointment under the Insurance Act, and we know of nothing that entitles him to be regarded as an authority on hospital administration, military or civil.

### INFIRMARY OFFICERS AND ENLISTMENT.

Metropolitan Boards of Guardians at the present time are face to face with a situation that they must meet in view of Lord Derby's appeal for men. In a great many instances the staffs have been reduced to the lowest minimum, and it is felt by the Guardians that they cannot allow any more men to go if they are to consider first the requirements of the sick patients under their care. At Lambeth it is reported that nineteen members of the male staff have joined the forces, and the board have come to the conclusion that they cannot dispense with any more men of serviceable age. They have indicated to the whole of the remaining medical staff, the engineers, and the ambulance-drivers that they cannot be spared from the Guardians' service, and have authorised the clerk to communicate this decision to the Parliamentary Recruiting Committee. At Wandsworth the steward at the infirmary wrote asking the Guardians for leave to join His Majesty's Forces. It was generally felt that his services were indispensable to the Guardians, who, however, were not desirous of placing any impediment in the way of an official of serviceable age enlisting. They decided to grant the steward the permission he asked for, although it was generally hoped that in view of his services to the public he would be exempt from Army service.

### BRADFORD WAR HOSPITAL.

The Local Government Board has approved of the proposal of the Guardians to establish a base hospital at St. Luke's Hospital, Bradford. The inmates of St. Luke's Hospital have been transferred to other similar institutions. The medical staff of the hospital will be as follows: Administrator, Mr. J. Phillips, with rank of lieut.-col.; registrar, Major D. Walker, R.A.M.C. (T.); senior resident physician, Dr. W. Wrangham; senior resident surgeon, Dr. W. S. Williamson, both with the rank of major. The visiting staff will be as follows:—Surgeons, Messrs. C. F. M. Althorp, Dr. Jason Wood, and Dr. W. Ward-Smith; physicians, Dr. H. J. Campbell and Dr. J. Beattie-Dunlop; radiographer, Dr. Wm. Mitchell. The arrangements have been practically settled by the War Office. The hospital will be administered entirely by the Guardians, and it is expected that the buildings will shortly be available for the reception of wounded soldiers. Messrs. Althorp, Wood, and Phillips and Drs. Campbell, Dunlop, Wrangham, and Mitchell are members of the honorary staff of the Bradford Royal Infirmary. Dr. Ward-Smith is honorary surgeon to Sir Titus Salt's Hospital, Saltaire, and to the Bradford Children's Hospital. Major Walker is a member of the Bradford City Council, and has been attached to the 6th West Riding Regiment.

### CAVELL MEMORIAL STATUE.

We are indebted to the courtesy of the *Daily Telegraph* for the information that money is coming in from all parts of the world for this statue and that the list is not yet closed. The *Daily Telegraph* originally asked for £2,000, but this sum is already exceeded, a circumstance which is welcome from the fact that the statue is to occupy a commanding site, and that, apart from the absorbing interest of the subject, it should be a work of art so exceptionally fine as to command attention on its merits.

### THE HOSPITAL OF FRIENDSHIP.

The institution having the above title went out to Belgium in January last, and was designed to provide accommodation for wounded during the frequent waits which they experience at railway junctions *en route* to the Base hospital. The idea was that a portable hospital, acting as a half-way house between the Front and the Base, might save much suffering. Therefore, as the plan received the approval of Inspecteur-Général Mélis, chief of the hospital arrangements of the Belgian Army, a committee, which included Lord Charles Beresford and Lady Bagot, was formed in London to carry it out. One lady provided the building, the Church Army most of the equipment, subscriptions came from friends, and two surgeons and their staff of ten men were soon on their way to Dunkirk. The actual building, which is made in sections, comprises a ward for twenty-six beds, 70 feet in length, a theatre, orderlies' room, and kitchen. The equipment includes a motor-ambulance. The committee also dispatched in May last a pavilion building as a waiting-room for slightly wounded men. Madame Curie, of radium fame, has been among the hospital's visitors.

### THE WAR'S EFFECT ON BEQUESTS TO CHARITY.

The depreciation in the value of securities caused by the war has led institutions to acquiesce in the postponement of legacies become due to them under recent wills. This is one of the odd results of the conflict, and an instance of the endorsement of this policy is to hand from Cambridge. At the recent quarterly court the committee announced that they had been notified of a legacy of £100 to Addenbrooke's Hospital, payable on the death of his wife, from Mr. E. J. Hall, of Newmarket. Owing to the war the trustees were advised not to realise the testator's investments. They proposed to retain the estate, both realty and personalty, in its present condition, and to receive the income arising from it. When the war is over the trustees propose to sell and divide the trust fund and the income accruing *pro rata* among the charitable legatees so far as the estate goes, minus expenses. The committee approved of the plan so far as Addenbrooke's was concerned.

### BADGES FOR PHARMACISTS?

A special meeting of the Bath Pharmaceutical Association was held lately to draw up a scheme of arrangements for the dispensing of medicines at the new war hospital by the members. Chemists had recently been canvassed as stretcher-bearers, but feeling they were likely to be more useful in their professional capacity had offered their services. Mr. E. Whiston is in charge of the scheme and is in daily attendance, while voluntary helpers are asked to give three hours' work once or twice a week. Two dispensers are required every morning, and two more at a later period of the day. To make their position quite clear, some of the chemists are inquiring whether or not there will be a badge which they may be allowed to wear. The matter apparently is in abeyance, pending the consideration of a letter from the chemists by the committee of the hospital, of which Lieut.-Colonel E. Lewis is chairman. As we have previously pointed out, badges for part-time service are not often favoured by the authorities, and not every part-time worker is eligible for wearing one. Have the Bath pharmacists really made out their case?

### A CHANGE OF POLICY.

Sir Arthur Downes, medical inspector of the Local Government Board, has notified the Metropolitan Asylums Board that no objection would be raised to the early provision of an operating-room at their South-Western Hospital for Infectious Diseases. In January last the managers approved of the provision of such a room at an estimated cost of £564, and whilst at the time the Local Government Board generally approved of the scheme, they were of opinion that it should be deferred for the time being. The withdrawal of their provisional veto may be noted by other Boards of Guardians, who have had similar schemes held up on the ground of economy, for it is no economy to defer a much-needed proposal that will eventually lead to economy and the better treatment of the sick.

### THE WORK OF A RURAL MEDICAL OFFICER.

Although Rutland ranks smallest among English counties in superficial area, its public health administration is far from being second-rate. Whatever may have been its record in the past, it is no exaggeration to describe it now as one of the best inspected rural districts in the kingdom, and the credit for this is due to the present county medical officer, Dr. C. Rolleston. His annual reports, of which the latest has just been printed, are evidence of the energy and pains taken by him to awaken the rural conscience in respect of the sanitary deficiencies which abound in Rutland as in numerous other country districts. Only those who are aware practically of the difficulties encountered by medical officers of health in rustic communities can fully appreciate the advances that have been made during the last five years in Rutland. An almost discouraging number of sanitary sins are still being committed, but by persistence and personal supervision a gratifying record of improvement is the result. In the matter of tuberculosis this county is served by an admirably adapted scheme well suited to the needs of the district. There has been erected on the county medical officer's premises a small dispensary, which in epuipment would stand comparison with many a more pretentious tuberculosis dispensary. A description of a similar rural dispensary, with details of its cost for building, furnishing, and its equipment, appeared in The Hospital for May 22, 1915. It is worth calling attention to the fact that the medical officer for Rutland receives a grant of only £40 a year for travelling in connection with tuberculosis alone, though he traverses by motor about 5,000 miles during the year. The allowance is decidedly not an extravagant one.

### A HOSPITAL SECRETARY AND THE NAVY.

We have been pleased to record the military service performed by many hospital secretaries since the outbreak of war, but, naturally enough, opportunities for service in the Navy have not been so numerous. It is therefore interesting to place on record the fact that a cottage hospital, the institution at Cromer, has contributed one of its most trusted officials to the Navy. At least the officer in question returned to his old love, for, as financial secretary to the hospital, Mr. George Bishop, R.N., had taken up charitable work after service at sea, and in June last he again returned to his ship. From its situation Cromer Cottage Hospital has been able to do much service for an institution of its size, and it is naturally proud of its connection with the Navy.

### HOSPITAL COMMITTEE AND COUNTY COUNCIL: A DISPUTED POINT.

A very nice point has been decided in the Court of Appeal, before the Master of the Rolls and Lord Justices Banks and Warrington, on a question arising out of the Isolation Hospitals Act of 1893. Acting under its provisions, the Derbyshire County Council constituted a hospital district and established a hospital committee to represent the different areas in the district and the County Council. On a disputed point, which arose later, the County Council, alleging powers under the Act, reconstituted the Hospital Committee in such a fashion as to secure the former a clear majority. A local authority, the Barlow and Bulwell, who originally had had one representative on the committee, thereupon brought an action against the County Council, the result of which was that Mr. Justice Sargant ruled that the order of the County Council was *ultra vires*. From this decision arose the present appeal. The Master of the Rolls, who admitted that the point was a difficult one, thought that Mr. Justice Sargant was right and that the County Council did not possess the unlimited powers that they claimed. The two other judges, however, took the view that the Hospital Committee was not intended to act independently of the County Council. The appeal, therefore, was allowed, and the action dismissed with costs.

### A MILITARY HOSPITAL MAGAZINE.

The *Craigleith Hospital Chronicle* maintains a healthy vitality, and its eleventh number is a most creditable one. Born last December, the *Chronicle* has not only provided reading of real worth, but has been the means of supplying extra comforts for the sick and wounded through the profits derived by its sale. The present number contains many well-written articles, including a bright little essay on the *estaminet*—the cosy café so helpful in cheering the Belgian, French, and English soldiers during the long evenings in Flanders. The author of this sketch has, we regret to learn, fallen in action. He was Lieut. J. C. Macpherson, of the 1st Gordon Highlanders. We trust that the *Chronicle* will continue its successful course, and we look forward with no small pleasure to the promised special and enlarged New Year's issue.

### SALARY OR FEES.

The Hatfield Board of Guardians is one of the very few which pay their district medical officers by fees instead of by a fixed salary. The Guardians wish to change the system, and fall into line with other Unions. The district medical officers are equally divided between opposition and indifference to the change. The fee system has the advantage that it ensures a definite relation between payment and work done. It is open to abuse by an unscrupulous medical officer; but such abuse is easy of detection if the Guardians do their work properly. The objection to it is that it may act as an undesirable check upon the granting of medical relief On the other hand, the salary system is often unjust to the Poor-Law medical officer, because of the absence of any such check. The fairest system would be one under which the district medical officer received a fixed salary, with an additional small fee for each case referred to him by the relieving officer.

### THE HEALTH OF HALIFAX.

The report for 1914 of the medical officer of health for Halifax, recently issued, contains some interesting items, as indeed do practically all the annual reports of large towns or country areas. In spite of "war marriages" the marriage rate declined to 9.9 per 1,000, an extraordinarily low rate. Similarly the birth-rate, which in 1914 cannot have been affected by the war, was 17.5, the lowest on record. No town in Yorkshire has such a low birth-rate, and even the few other towns which compete with Halifax in this respect have the excuse of a large unmarriageable population (invalids and old people in the cases of Bath, Bournemouth, Eastbourne, Hastings, Southend, Southport, Blackpool, and undergraduates in the cases of Oxford and Cambridge). The death-rate also fell, but the surplus of births over deaths is at a steadily declining figure in this town. The infant mortality stands at 103 per 1,000 births. This is not a high figure as our large towns go, but Halifax has shown a smaller death-rate three times in the previous five years, and it is by no means an ideal ratio. It is clear that the M.O.H. is active in promoting remedial measures, but the people of Halifax should not rest content with the statistics which he records.

### MOTOR AMBULANCE FOR THE NAVY.

A motor ambulance has been bought for the Navy by the Council of the St. Andrew's Ambulance Association, and was formally presented to Surgeon-General M'Nab, R.N., by Colonel D. J. Mackintosh, M.B., M.V.O., chairman of the Council, at the head offices of the Association in Glasgow. The waggon has been provided by subscription among the members of the Council and their friends. In making the presentation Colonel Mackintosh dwelt with justifiable pride upon the services which the St. Andrew's Association has rendered to the Army since the outbreak of war, in conjunction with the Transport Department of the Scottish Branch of the Red Cross Society. Now, by handing over a motor ambulance they are in some degree assisting in the work of the Navy; and there is a special appropriateness in the ceremony taking place in the foremost seaport of Scotland, a fact which Surgeon-General M'Nab evidently realised in a felicitous speech of thanks on behalf of the Lords Commissioners of the Navy.

### THIS WEEK'S DRUG MARKET.

The scarcity of some of the commonly prescribed synthetic drugs has now reached such an acute stage that it is difficult to obtain even small supplies of them, and there is the possibility that soon several of these products will become unobtainable at any price. Phenazone has again advanced in price very substantially, and has become much scarcer. Phenacetin, barbitone, and trional are also dearer. Quinine is still quoted at what under the circumstances can only be regarded as a high price, and there certainly seems ground for the belief that the price will decrease; this applies to the Continental brands, in which such a large business was recently done by speculators and American buyers, but British makers still quote a figure that may be regarded as reasonable, although they are not, of course, free sellers. Ipecacuanha is selling at very high prices, the reason being the large demand for this material for the purpose of manufacturing emetine for the treatment of dysentery. The value of opium is well maintained. There is practically no change in the position of bromides, which are still quoted at extremely high rates.

